# Amoebae can promote the survival of *Francisella* species in the aquatic environment

**DOI:** 10.1080/22221751.2021.1885999

**Published:** 2021-02-24

**Authors:** Aurélie Hennebique, Julien Peyroux, Camille Brunet, Amandine Martin, Thomas Henry, Masa Knezevic, Marina Santic, Sandrine Boisset, Max Maurin

**Affiliations:** a Service de Bactériologie-Hygiène Hospitalière, Centre National de Référence des Francisella, Institut de Biologie et de Pathologie, Centre Hospitalier Universitaire Grenoble Alpes, Grenoble, France; b Université Grenoble Alpes, Centre National de la Recherche Scientifique, Grenoble, France; c CIRI, Centre International de Recherche en Infectiologie, Lyon, France; d Faculty of Medicine, Department of Microbiology and Parasitology, University of Rijeka, Rijeka, Croatia

**Keywords:** *Francisella*, *Francisella tularensis*, amoebae, tularemia, microbial ecology

## Abstract

*Francisella tularensis*, a tier 1 select agent, is the causative bacterium of tularemia, a zoonosis with a large animal reservoir. However, *F. tularensis*, like many other *Francisella* species, is assumed to have an aquatic reservoir. The mechanisms of *Francisella* species persistence in surface water remain poorly characterized. In this study, we deeply investigated the long-term interactions of the tularemia agent *F. tularensis* subsp. *holarctica*, *F. novicida* or *F. philomiragia* with amoebae of the *Acanthamoeba* species. In amoeba plate screening tests, all the *Francisella* species tested resisted the attack by amoebae. In *in vitro* infection models, intra-amoebic growth of *Francisella* varied according to the involved bacterial species and strains, but also the amoeba culture medium used. In co-culture models, the amoebae favoured *Francisella* survival over 16 days, which was likely dependent on direct contact between bacteria and amoebae for *F. novicida* and on amoeba-excreted compounds for *F. novicida* and for *F. tularensis*. In a spring water co-culture model, amoebae again enhanced *F. novicida* survival and preserved bacterial morphology. Overall, our results demonstrate that amoebae likely promote *Francisella* survival in aquatic environments, including the tularemia agent *F. tularensis*. However, bacteria-amoebae interactions are complex and depend on the *Francisella* species considered.

## Introduction

*Francisella tularensis* is a Gram-negative, facultative intracellular bacterium, causing the potentially life-threatening zoonosis tularemia. This microorganism is classified as a category A potential agent of biological threat by the US Centers for Disease Control and Prevention (CDC) [1]. Taxonomically, the *F. tularensis* species is divided into four subspecies. *F. tularensis* subsp. *tularensis* (Type A) and *F. tularensis* subsp. *holarctica* (Type B) are the etiological agents of tularemia. *F. tularensis* subsp*. mediasiatica* is restricted to central Asia and Russia, and has never been isolated from humans. *F. tularensis* subsp. *novicida* is an aquatic bacterium of low virulence in humans, also considered a different species, *F. novicida* [2,3]. For clarity, in our study we will differentiate *F.* tularensis (type A and type B tularemia agents) from the aquatic bacterium *F. novicida*. *F. tularensis* can infect a wide range of animals, which constitute the primary reservoir of this species [4]. Ticks and other arthropods (especially mosquitoes) are vectors of this pathogen [4]. More recently, the hydro-telluric environment has been highlighted as a probable major reservoir of this zoonotic agent and as a source of human infections [5]. Although *F. tularensis* has been detected in natural water sources [5], the role of the aquatic environment as a reservoir of this species remains to be firmly established. Furthermore, the mechanisms and conditions of *F. tularensis* long-term survival in this environment have to be characterized. Some intracellular bacteria such as *Legionella pneumophila* and *Mycobacterium avium* persist in natural ecosystem by using free living amoebae as a multiplication niche and reservoir [6]. Amoebae such as *Acanthamoeba* and *Vermamoeba* species are ubiquitous in water and soil [7]. A few experimental studies have investigated the relationships between *Francisella* sp. and amoebae, with contradictory results [8–14]. Most of these studies have involved the live vaccine strain (LVS) of *F. tularensis*, *F. novicida*, *F. philomiragia*, or *F. noatunensis* [8–14]. Only two studies have evaluated the interactions with amoebae of virulent *F. tularensis* type A strains [8,14] and only one with a virulent *F. tularensis* type B strain [14].

In mammalian cells, it is well established that *Francisella* species multiplies intracellularly thanks to an atypical Type VI secretion system (T6SS) encoded in the *Francisella* Pathogenicity Island (FPI) [15–17]. *F. tularensis* possesses two almost identical copies of the FPI in its genome, while *F. novicida* and *F. philomiragia* only possess a single copy [17,18]. *F. novicida* harbours another genetic locus, that we termed FNI, likely encoding a different T6SS of unknown function [17,19]. The role of *Francisella* T6SSs in persistence/replication within amoebae remains unclear [9,11,12,20].

In the present study, we conducted a detailed characterization of the interactions of three *Francisella* species, including a virulent type B strain, with two amoebic species in order to further characterize the potential role of amoebae in the long-term survival of these species in the aquatic environment.

## Materials and methods

### Bacterial strains

The following strains of *Francisella* sp. were used (Table S1): the reference strains *F. philomiragia* ATCC 25015, *F. novicida* U112 (CIP 56.12), and *F. tularensis* subsp. *holarctica* LVS NCTC 10857; one clinical strain of *F. philomiragia* (Ft47); six clinical strains of *F. tularensis* subsp. *holarctica* (Ft5, Ft6, Ft7, Ft46, Ft62, Ft74), *F. novicida* ΔFPI mutant [21], *F. novicida* ΔFNI mutant [19], and the *F. novicida* ΔFPIΔFNI double mutant. Clinical strains of *F. tularensis* subsp. *holarctica* were isolated from French patients suffering from the different tularemia forms. It was previously shown that all French clinical strains belong to clade B.44 and display low genetic diversity (genomes sequences of these clinical strains are available at DDBJ/EMBL/GenBank under the BioProject number PRJNA551589) [22]. Therefore, for most experiments, the Ft6 strain was used as representative of clinical strains. Clinical strain of *F. philomiragia* (Ft47) was isolated from a French patient suffering from a systemic infection with bacteraemia. All *Francisella* strains used in this study are owned by the French National Reference Center for *Francisella* (Grenoble University Hospital, Grenoble, France). Specific authorizations were obtained from the Agence Nationale de Sécurité du Médicament et des produits de santé (ANSM, authorization number ADE-103892019-7) for *F. tularensis* strains. For experiments, *Francisella* sp. strains were grown on chocolate agar media supplemented with PolyViteX® (bioMérieux, Marcy-l’Etoile, France), incubated at 35°C in a 5% CO_2_-enriched atmosphere for one day for *F. philomiragia* and *F. novicida* strains, and two days for *F. tularensis* strains. Experiments were all conducted in a biosafety level 3 (BSL3) laboratory. The *F. novicida* ΔFPIΔFNI double mutant (U112 Δ*FTN_1309-1325::aphA,* Δ*FTN_0037-0054::FRTsc*) was generated in the wild-type U112 strain by transforming the ΔFNI mutant with genomic DNA from the ΔFPI mutant using chemical transformation. Briefly, the ΔFNI mutant grown in Tryptic Soy Broth (TSB), 0.1% L-cys and 0.4% glucose until an O.D.600 nm of 0.9 was washed once and 10-fold concentrated in chemical transformation buffer (Tris 50 mM, NaCl 270 mM, MgSO4, 25 mM, CaCl2, 20 mM, L-Arg 2 mM, L-His 1 mM, L-Met 2 mM, L-Asp 3 mM, Spermine 0.2 mM; MnCl2 35 mM, pH6.8). 500 μl of the obtained bacterial solution was incubated for 20 min at 37°C with shaking 100 rpm with 1 µg gDNA from the ΔFPI strain (Δ*FTN_1309-1325::aphA).* 1 ml TSB, 0.1% L-cys and 0.4% glucose was then added to the bacteria which were further incubated for 2 h at 37°C before plating on selective plates (Tryptic Soy Agar (TSA), 0.1% L-cys, 10 μg/ml Kanamycin). Following clonal isolation, gene deletions were verified by PCR on gDNA using FPI and FNI flanking primers. Other bacterial strains used as controls included *Staphylococcus epidermidis* ATCC 1228 and *Legionella pneumophila* CIP107629 T. *S. epidermidis* ATCC 1228 was grown on sheep blood agar medium (bioMérieux, Marcy-l’Etoile, France) and *L. pneumophila* on Buffered Charcoal Yeast Extract (BCYE) medium (Oxoid, Cambridge, UK).

### Amoebae and amoebae culture media

We used *Acanthamoeba castellanii* (Neff) and *A. polyphaga* (Linc-AP1) strains. Amoebae were grown axenically in peptone-yeast extract-glucose medium (PYG, Eurobio, France) at 27°C, in 75-cm^2^ cell culture flasks (Falcon, Corning Incorporated, Life Sciences, Durham, USA). Peptone-yeast extract-glucose medium (PYG = ATCC medium 712) is a rich culture medium (2% proteose peptone, 0.1% yeast extract, 0.1 M glucose, 4 mM MgSO_4_ - 7H_2_O, 0.4 mM CaCl_2_, 0.1% sodium citrate dihydrate, 0.05 mM Fe(NH_4_)_2_(SO_4_)_2_ - 6H_2_O, 2.5 mM Na_2_HPO_4_ - 7H_2_O, 2.5 mM KH_2_PO_4_) supporting *Acanthamoeba* sp. living in trophozoite form and multiplication.

For experiments, several amoebae culture media were used. PYG without glucose medium (PYG/wg) presents the same composition as PYG medium except that it does not contain glucose. This medium supports *Acanthamoeba* sp. survival in trophozoite form for 16 days but does not allow amoebae multiplication (data not shown). Starvation medium (SM) is a less nutritive medium (1 mM Na_2_HPO_4_, 1 mM KH_2_PO_4_, 0.016 mM MgSO_4_ - 7H_2_O, 0.027 mM CaCl_2_ - 2H_2_O, 2 mM NaCl, 0.005 mM (NH_4_)_2_Fe(SO_4_)_2_ - 6H_2_O, 0.2% yeast extract, 1,8% glucose) [23] allowing *Acanthamoeba* sp. survival in trophozoite form for 16 days without amoebae multiplication (data not shown). Page's amoeba saline (PAS = ATCC medium 1323) is a poor medium (1 mM Na_2_HPO_4_, 1 mM KH_2_PO_4_, 0.016 mM MgSO_4_ - 7H_2_O, 0.027 mM CaCl_2_ - 2H_2_O, 2 mM NaCl) allowing *Acanthamoeba* sp. survival in cystic form without multiplication.

### Amoeba plate tests (APT)

This test allows evaluation of interactions of bacteria with an amoebae monolayer established on the surface of an agar plate [10,24]. APT were performed using *A. castellanii* or *A. polyphaga*, for *F. tularensis* (LVS, and clinical Ft5, Ft6, Ft7, Ft46, Ft62, and Ft74 type B strains), *F. philomiragia* (ATCC 25015, or Ft47 clinical strain), and *F. novicida* U112.

Amoebae grown in PYG medium in 75-cm^2^ cell culture flask were harvested at 90% confluence by rapping the flask to bring amoebae into suspension. Amoebae were counted, centrifuged 10 min at 1000 g, and resuspended in fresh PYG medium at a concentration of 2.67 × 10^6^ cells/ml. The amoebae suspension (1.5 ml) was spread on chocolate agar plates and allowed to dry 1-2 h to form an amoebae monolayer on the surface of the agar medium. For each bacterial strain tested, a suspension of 10^9^ CFU/ml was prepared in Phosphate-Buffered Saline (PBS, Thermo Fisher Scientific). Tenfold serial dilutions (from 10^9^ to 10^6^ CFU/ml) of this suspension were prepared, and 10 µl of each dilution was inoculated on the amoebae monolayer. The agar plates were then incubated at 30°C for 10 days, and examined daily for bacterial growth. As a bacterial growth control, chocolate agar plates without amoeba were inoculated with the same bacterial suspensions. For negative and positive APT controls, *S. epidermidis* ATCC 1228 and *L. pneumophila* CIP107629 T respectively were inoculated to amoebae monolayers using the same protocol except that BCYE agar plates were used for *L. pneumophila.* For *Francisella* strains, the results of APTs were evaluated visually and semi-quantitatively according to the lowest bacterial inoculum allowing colony formation and the intensity of the bacterial growth (equal or inferior) in comparison to the amoeba-free growth control. *Francisella* growth on the amoeba monolayer was also compared to that of *L. pneumophila* on the same protozoa. Experiments were performed twice.

### Growth of *Francisella* strains in amoebae culture media

We tested the capacity of *F. philomiragia* ATCC 25015, *F. novicida* U112, and the LVS and Ft6 strains of *F. tularensis* to grow in the amoebae culture media PYG, PYG/wg, SM, and PAS, in the absence of amoeba. Each medium was inoculated with 10^5^ CFU/ml of the tested bacterial strain, spread in 24-well plates and then incubated at 27°C. At different time intervals over a 16-day period, an aliquot of each culture medium was sampled, serially diluted and spread onto chocolate agar plates to determine CFU counts. Experiments were performed twice, each time in triplicate.

### Amoebae infection with *Francisella* models

We evaluated intra-amoebic growth of *Francisella* strains (*F. philomiragia* ATCC 25015, *F. novicida* U112, and *F. tularensis* LVS and Ft6 strains) in *A. castellanii* or *A. polyphaga*. Two models were evaluated either using PYG/wg or SM media as the amoebae culture supernatant.

For the first model, a suspension of 5.3 × 10^5^ cells/ml in PYG/wg medium was prepared for each amoeba strain tested, using the same procedure described for APT experiments. Then, the amoebae suspensions were dispensed in five 24-well plates (950 µL per well, each plate containing five wells for each amoeba strain), and amoebae were allowed to adhere for 1 h at 27°C. For each *Francisella* sp. strain tested, a bacterial suspension (50 µl of 10^8^ CFU/ml in PYG/wg) was added to wells containing either *A. castellanii* or *A. polyphaga* (in triplicate) to obtain a multiplicity of infection (MOI) of 10. The plates were centrifuged at 1000 g for 20 min to optimize contact between bacteria and amoebae, and incubated 40 min at 27°C. Then, the culture supernatant was removed, the amoebae layer was washed three times with PAS, and new PYG/wg medium containing 100 µg/ml of gentamicin was added to remove non-phagocytized bacteria. The plates were further incubated 1 h at 27°C. The amoebae monolayers where then washed three times with PAS and finally incubated in new PYG/wg medium at 27°C. This time of the experiment corresponded to T0. Plates were incubated during seven days at 27°C. CFU counts were determined at T0, day one (D1), D2, D5 and D7 of incubation of cell cultures (one plate per each time). Therefore, the culture supernatant of each well was removed and amoebae were lysed with 1% saponin for 10 min at 27°C followed by vigorous pipetting. The amoebic lysate was serially diluted and spread onto chocolate agar plates to determine intra-amoebic CFU counts. In parallel, the removed supernatant was also serially diluted and spread onto chocolate agar plates to determine extra-amoebic CFU counts. The bacterial detection limit was 10 CFU/ml.

For the second model, the same procedure as above was performed, but SM was used instead of PYG/wg and extra-amoebic CFU counts were not determined.

Experiments were performed twice, each time in triplicate.

### Amoebae and *Francisella* co-culture models

We developed two co-culture models to evaluate growth of *Francisella* strains in the presence of amoebae, with or without direct contact of bacteria with these protozoa.

In the first model, *A. castellanii* or *A. polyphaga* were infected with *F. philomiragia* ATCC 25015, *F. novicida* U112, or *F. tularensis* Ft6 strains, using the same procedure as above and SM as the amoeba culture medium. However, killing of non-phagocytized bacteria using gentamicin was not performed. The plates were incubated at 27°C for 16 days. At T0, D2, D7, D12 and D16, amoebae were lysed by addition of 1 ml of 2% saponin during 10 min at 27°C and vigorous pipetting without removing the amoebae supernatant. The lysates were serially diluted and spread onto chocolate agar plates in order to determine the total (i.e. intra- and extra-amoebic) CFU counts. Control wells without amoeba were prepared using the same protocol. The bacterial detection limit was 20 CFU/ml.

In the second model, co-culture conditions were the same as above, but bacteria and amoebae were separated by a cell culture insert with a pore size of 0.4 µm (Millicell Merck Millipore, Darmstadt, Germany). Therefore, there was no direct contact between these microorganisms, but possible interactions due to a shared culture medium. In co-culture with inserts model, at each time point listed previously, supernatant above the insert was sampled, serially diluted and spread onto chocolate agar plates in order to determine CFU counts. After this step, insert was removed and 1 ml of 2% saponin was added on the amoebae supernatant for 10 min at 27°C followed by vigorous pipetting. The lysate was spread onto chocolate agar plate in order to check for the absence of bacteria.

Experiments were performed twice, each time in triplicate.

### Amoebae survival

During infection and co-culture experiments, wells with infected or uninfected amoebae were incubated in similar conditions. At each time point, amoebic morphology and adherence of infected and uninfected amoebae were checked under a microscope. After amoebae detachment through vigorous pipetting, determination of the percentage of mortality were performed using trypan blue dye in a counting chamber. In addition, for co-culture experiments that lasted for 16 days, total amoebae counting was also performed throughout the experiment.

### Immunofluoresence detection of *Francisella* in infected amoebae

At different time points of the infection experiments in PYG/wg or SM media, infection of amoebae with *Francisella* strains was evaluated by immunofluorescence and confocal microscopy. Infection experiments were performed as described above in 24-well plates, except that a 12 mm microscopy slide (SPL Life Sciences, Korea) was added in each well at the beginning of the experiments. At the different incubation times previously indicated, microscopy slides were removed from wells, fixed with 3.7% paraformaldehyde for 20 min at room temperature and then washed with PBS. Amoebae were permeabilized with PBS-Triton X-100 0.2% for 10 min at room temperature followed by two PBS washes. Blocking was performed with 3% bovine serum albumin for 30 min at room temperature. Rabbit anti-*Francisella tularensis* primary antibody (# TC-7005, Tetracore, Rockville, USA) diluted 1:100 in PBS-BSA 0.3% was added for 1 h in humid chamber at room temperature followed by three washes with PBS-BSA 0.3%-Tween 0.01%. This anti-*Francisella tularensis* antibody was shown to also label *F. philomiragia* (data not shown). Then, AlexaFluor 594 goat-anti-rabbit secondary antibody (# A-11012, Invitrogen, Thermo Fisher Scientific, Rockford, USA) diluted 1:1000 in PBS-BSA 0.3%, together with AlexaFluor 488 phalloidin (Invitrogen, Thermo Fisher Scientific, Rockford, USA) were added for 1 h at obscurity in a humid chamber at room temperature, respectively for *Francisella* and amoebae actin staining. Slides were then washed with PBS-BSA 0.3%-Tween 0.01% twice, then with PBS twice. They were mounted with ProLong Glass (Invitrogen, Thermo Fisher Scientific, Rockford, USA) and examined by immunofluorescent microscopy (on a Nikon Eclipse TS100) and confocal microscopy. Confocal microscopy was performed using a confocal laser scanning microscope (CLSM, LSM710, Zeiss, Jena, Germany) equipped with a Plan-Apochromat 63×/1.40 Oil DIC M27 objective.

### Study of the T6SS impact on *Francisella* and amoebae interactions

The role of the *F. novicida* T6SS in amoebae interactions was evaluated using the previously described models. APT were performed for *A. castellanii* and either wild-type (WT) *F. novicida* U112, *F. novicida* ΔFPI, *F. novicida* ΔFNI, or *F. novicida* ΔFPIΔFNI. Infections in PYG/wg and SM were performed for *A. polyphaga* and either WT *F. novicida* U112 or *F. novicida* ΔFPIΔFNI. Co-culture without inserts were performed for *A. polyphaga* and WT *F. novicida* U112 or *F. novicida* ΔFPIΔFNI. Experiments were performed twice, each time in triplicate.

### Amoebae and *Francisella* co-cultures in spring water

Spring water (pH 7.5) was collected from a large permanent spring in Croatia and then autoclaved at 121°C for 15 min. *F. novicida* U112 and *A. castellanii* were inoculated in 1 L of spring water in glass bottles, with shaking, and capped with cap half-loosened, at room temperature. Amoebae and bacteria concentrations were of: 10^6^ *F. novicida*/ml alone, 10^7^ *F. novicida*/ml alone, 10^6^ *F. novicida*/ml together with 10^7^ *A. castellanii*/ml, 10^7^ *F. novicida*/ml together with 10^6^ *A. castellanii/*ml, 10^6^ *A. castellanii*/ml alone and 10^7^ *A. castellanii/*ml alone. Two hours after inoculation, most of the amoebae were adhered to the bottle, the rest were floating in the water. Growth kinetics of bacteria and amoebae were followed every five days in a period of 30 days. The suspensions were homogenized by shaking before each sampling. The number of *F. novicida* at each time point was determined by plating serial dilutions on BCYE agar plates. In order to determine the number of amoeba cells, at each time point 1 ml of water sample was transfered to a 24 well plate, and left for 2 h at room temperature to allow amoebae to adhere. It was followed by analysing the samples using light microscopy. Experiments were performed twice, each time in triplicate.

During these experiments in spring water, morphology and structure of *F. novicida*, after 15 days incubation alone in spring water or in co-incubation with *A. castellanii* in spring water was studied by TEM in comparison to control bacteria grown on BCYE agar. Bacteria were prepared for TEM by negative staining. Bacterial suspension was applied to the Carbon Coated 200 mesh Cooper Grid for 2 min, and drown off from the edge of the grid with filter paper. After that, the grid was stained using 10 µl of 2% phosphotungstic acid for 1 min and again drained with the filter paper. The grid was placed directly into the grid box and allowed to air dry before observation. By transmission electron microscopy on a Zeiss 902A, we observed morphology of bacteria, including their size, shape and density. Ten fields for each sample were randomly photographed.

### Statistical analysis

A two-tailed Student t-test was used to compare bacterial loads in the different experimental models, using a significance level of 0.05.

## Results

### Amoeba plate tests

The APT was first performed as a screening assay of interactions between a monolayer of amoebae and *Francisella* sp strains. Figure S1 shows some visual results of the APT tests. All *Francisella* strains tested were able to pass through the amoebae monolayer forming bacterial colonies, demonstrating resistance of these bacteria to the predatory properties of amoebae. *F. philomiragia* (ATCC 25015 and Ft47) growth was observed at the 10^6^ CFU/ml dilution with the same intensity with or without amoebae and similar to the *L. pneumophila* control. For the clinical strains of *F. tularensis*, and *F. novicida* U112, growth was observed at the 10^6^ CFU/ml dilution with or without amoebae but with inferior intensity in the presence of amoebae and compared to the *L. pneumophila* control. Finally, for the LVS strain, growth on the amoebae monolayer was observed at the 10^9^ CFU/ml dilution (3 log inferior to the amoeba-free control and the *L. pneumophila* control).

### Growth of *Francisella* strains in amoebae culture media

In order to define the most suitable supernatant medium for further evaluations of *Francisella* and amoebae interactions, we evaluated the growth of *F. tularensis* LVS and Ft6 strains, *F. philomiragia* ATCC 25015, and *F. novicida* U112 in the amoebae culture media PYG, PYG without glucose (PYG/wg), starvation medium (SM), and PAS. In the absence of amoeba, the four bacterial strains showed strong growth in PYG and PYG/wg media, with an increase of more than 4 log of CFU counts after 16 days of incubation of cultures (Figure S2). In contrast, these strains were unable to grow in SM or PAS media, with a progressive decrease in CFU counts up to undetectable level within 5–7 days for PAS and 5–16 days for SM (Figure S2).

### Amoebae infection with *Francisella* in PYG/wg

Since APT revealed interactions between *Francisella* sp. and amoebae, we needed to better characterize these interactions. In this aim, we first used an infection model in a rich culture medium (i.e. PYG/wg) previously described in the literature [9,11] but with longer incubation period. Infections of *A. castellanii* or *A. polyphaga* with *F. philomiragia* ATCC 25015, *F. novicida* U112, or *F. tularensis* (LVS or Ft6 strains) were performed.

**Figure 1.**
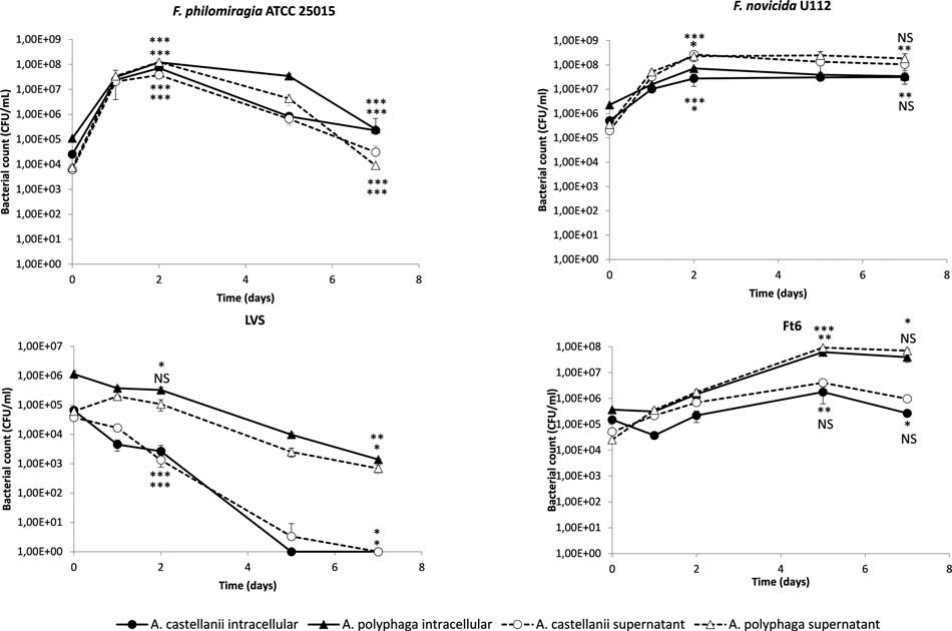
Infection of *A. castellanii* and *A. polyphaga* by *Francisella* sp. in PYG/wg. The figure shows one experiment made in triplicate. Similar results were obtained in a second experiment. The error bars represent standard deviations. Comparison of T0 versus D2 (or D5 for Ft6), and D2 (or D5 for Ft6) versus D7. NS: not significant; *: *p* < 0.05; **: *p* < 0.01; ***: *p* < 0.001.

During the first two days of incubation of cultures, we observed a significant increase in intracellular CFU counts for *F. philomiragia* in *A. polyphaga* (3.04 log CFU/ml, *p* < 0.01) and *A. castellanii* (3.46 log CFU/ml, *p* < 0.001) and for *F. novicida* in *A. polyphaga* (1.50 log CFU/ml, *p* < 0.001) and *A. castellanii* (1.73 log CFU/ml, *p* < 0.05). Then, a progressive decrease (for *F. philomiragia*) or stagnation (for *F. novicida*) of intra-amoebic CFU counts was observed the following five days. CFU counts of both *Francisella* strains in the amoebae culture supernatant evolved similarly to the intra-amoebic CFU counts with difference between them at each time point of one log or less (Figure 1).

As for *F. tularensis* Ft6, we observed a weaker and slower increase in CFU counts within the amoebic compartment, with a peak at five days of incubation, also more marked for *A. polyphaga* (2.22 log CFU/ml, *p* < 0.01) than for *A. castellanii* (1.07 log CFU/ml, not significant (NS)). Here again, CFU counts in culture supernatant evolved similarly to the intra-amoebic CFU counts with less than one log difference between them at each time point (Figure 1).

In contrast, the LVS strain did not display any intra- or extra-amoebic growth, but a progressive decline in intra-amoebic CFU counts during the seven days of the experiments, of 2.91 log CFU/ml (*p* < 0.01) for *A. polyphaga* and to undetectable level for *A. castellanii* (*p* < 0.001) (Figure 1).

For the four bacterial strains tested, microscopic observations of infected and uninfected amoebae showed conservation of adherence and maintenance in trophozoite form of these protozoa at each time point of the experiments and amoebae mortality rates were inferior to 10% (data not shown).

### Amoebae infection with *Francisella* in SM

In the absence of amoebae, we observed that PYG/wg supported a progressive growth of *Francisella* strains over time, whereas SM did not. Indeed, to prevent any extracellular growth of *Francisella* that may interfere with intra-amoebic CFU counts monitoring, we then developed an infection model in poor culture medium (i.e. SM), precluding extracellular growth of these bacteria. *A. castellanii* or *A. polyphaga* were infected with *F. philomiragia* ATCC 25015, *F. novicida* U112, or *F. tularensis* (LVS or Ft6 strains) using the same procedure as above but SM as the culture supernatant.

**Figure 2.**
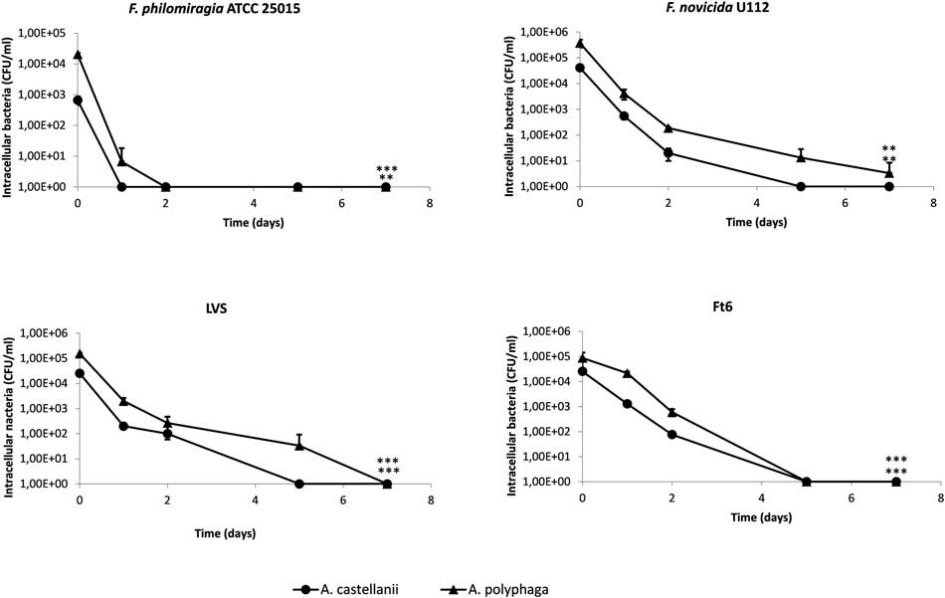
Infection of *A. castellanii* and *A. polyphaga* by *Francisella* sp. in SM. The figure shows one experiment made in triplicate. Similar results were obtained in a second experiment. The error bars represent standard deviations. Comparison of T0 versus D7. NS: not significant; *: *p* < 0.05; **: *p* < 0.01; ***: *p* < 0.001.

In this model, a rapid decrease of intra-amoebic CFU counts was observed up to undetectable level within one to seven days of incubation of cultures depending on the *Francisella* sp. strain considered (Figure 2).

For the four bacterial strains tested, microscopic observations of infected and uninfected amoebae showed conservation of adherence and maintenance in trophozoite form of these protozoa at each time point of the experiments and amoebae mortality rates were inferior to 10% (data not shown).

### Amoebae and *Francisella* co-cultures in SM

Since APT revealed that *Francisella* sp. resisted to amoebae and infection in SM did not suggested intra-amoebic survival of *Francisella* sp., we developed co-culture models in order to evaluate if amoebae could promote *Francisella* sp. survival whether intracellular or extracellular. In co-culture model, after the infection step, extra-cellular bacteria were not killed. This model was supposed to be closer to the interactions between amoebae and bacteria that may occur in nature. Co-cultures of *A. castellanii* or *A. polyphaga* with *F. tularensis* Ft6, *F. philomiragia* ATCC 25015 or *F. novicida* U112 were established using SM as the amoebae culture medium.

**Figure 3.**
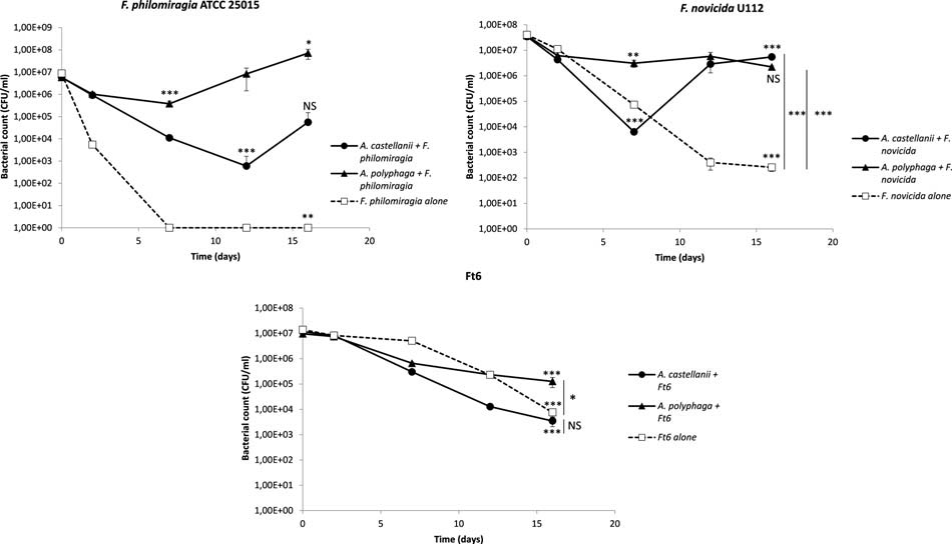
Co-culture of *A. castellanii* and *A. polyphaga* with *Francisella* sp. in SM. The figure shows one experiment made in triplicate. Similar results were obtained in a second experiment. The error bars represent standard deviations. Comparison of T0 versus D7, D12 or D16; or D16 versus D7 or D12. Comparison of bacteria with amoebae and bacteria alone at D16. NS: not significant; *: *p* < 0.05; **: *p* < 0.01; ***: *p* < 0.001.

We observed an enhanced survival of both *F. philomiragia* and *F. novicida* in the presence of *A. castellanii* or *A. polyphaga*, compared to amoeba-free SM medium (Figure 3). The *F. philomiragia* and *F. novicida* CFU counts declined during the first seven to 12 days of experiments, both in the presence of *A. castellanii* (3.99 log CFU/ml, *p* < 0.001; and 3.73 log CFU/ml, *p* < 0.001, respectively) or *A. polyphaga* (1.19 log CFU/ml, *p* < 0.001; and 1.08 log CFU/ml, *p* < 0.01, respectively) (Figure 3). However, we then observed an increase in *F. philomiragia* CFU counts between days 12 and 16 for *A. castellanii* (1.97 log CFU/ml, NS), and days 7 and 16 for *A. polyphaga* (2.28 log CFU/ml, *p* < 0.05). Between days 7 and 16, *F. novicida* CFU counts significantly increased in *A. castellanii* (2.93 log CFU/ml, *p* < 0.001), and remained relatively stable in *A. polyphaga* (Figure 3). In contrast, CFU counts steadily and significantly decreased in the absence of amoebae, to undetectable level for *F. philomiragia* (*p* < 0.01), and by 5.19 log CFU/ml (*p* < 0.001) after 16 days for *F. novicida* (Figure 3). Therefore, there was a clear persistence of both *F. philomiragia* and *F. novicida* in the presence of either amoeba species, compared to amoeba-free culture conditions. For *F. novicida*, the difference in CFU counts at D16 was 4.32 log CFU/ml (*p* < 0.001) between amoeba-free and *A. castellanii* cultures, and 3.93 log CFU/ml (*p* < 0.001) between amoeba-free and *A. polyphaga* cultures. For *F. philomiragia*, the difference in CFU counts between amoeba-free and either *A. castellanii* or *A. polyphaga* cultures was more than four log (p could not be evaluated).

For *F. tularensis* Ft6, CFU counts progressively and similarly decreased between T0 and D16 in the presence or absence of amoebae: 3.53 log CFU/ml reduction (*p* < 0.001) in the presence of *A. castellanii*; 1.88 log CFU/ml (*p* < 0.001) with *A. polyphaga*; and 3.27 CFU/ml (*p* < 0.001) without amoebae (Figure 3). At day 16 of incubation, CFU counts were not statistically different between Ft6 alone and Ft6 with *A. castellanii* and was slightly superior for Ft6 with *A. polyphaga* than for Ft6 alone (*p* < 0.05) (Figure 3).

For the three bacterial strains tested, microscopic observations of infected and uninfected amoebae showed conservation of adherence and maintenance in trophozoite form of these protozoa. Total amoebae counts varied from 3 × 10^5^–8 × 10^5^ amoebae/ml and amoebae mortality rates were inferior to 10% (data not shown).

### Amoebae and *Francisella* co-cultures in SM in the presence of inserts

We tried to further investigate the mechanisms implicated in the enhanced survival of *F. novicida* U112 and *F. philomiragia* ATCC 25015 in co-culture with amoebae. In this aim, we established new co-cultures of these species in the presence of *A. castellanii* or *A. polyphaga*. However, direct contact between bacteria and amoebae was prevented by using cell culture inserts, while these microorganisms still shared the same culture medium.

**Figure 4.**
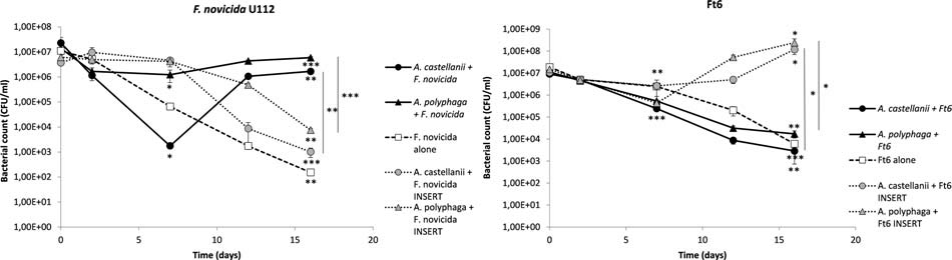
Co-culture with inserts of *A. castellanii* and *A. polyphaga* with *Francisella* sp. in SM. The figure shows one experiment made in triplicate. Similar results were obtained in a second experiment. The error bars represent standard deviations. Comparison of T0 versus D7 or D16; or D7 vs D16. Comparison of bacteria with amoebae separated or not by an insert at D16. NS: not significant; *: *p* < 0.05; **: *p* < 0.01; ***: *p* < 0.001.

Enhanced survival of *F. novicida* in the presence of either of the two amoebae species was partially lost when bacteria and amoebae were separated by an insert. *F. novicida* CFU counts progressively decreased between T0 and D16 in wells with inserts (3.56 log CFU/ml reduction (*p* < 0.001) for *A. castellanii* and 2.91 CFU/ml reduction (*p* < 0.01) for *A. polyphaga*) (Figure 4). For each amoeba species, CFU counts in wells with versus without inserts were significantly different at D16 (*p* < 0.01 and *p* < 0.001 for *A. castellanii* and *A. polyphaga,* respectively) (Figure 4). However, bacterial counts at D16 in wells with amoebae and inserts were significantly higher than in wells with bacteria alone (*p* < 0.05 for *A. castellanii* and inserts and *p* < 0.001 *A. polyphaga* and inserts).

*F. philomiragia* ATCC 25015 co-culture experiments with inserts gave non reproducible results within and between experiments and thus were not included in this study.

Although Ft6 survival was not favoured by the presence of amoebae, we performed co-cultures with insert for this bacterial strain. Surprisingly, *F. tularensis* Ft6 better survived when it was separated from the amoebae compared to co-cultures without inserts. When bacteria were separated from amoebae by an insert, CFU counts decreased during the first seven days (0.61 log CFU/ml (*p* < 0.01) reduction for *A. castellanii* and 1.54 log CFU/ml reduction (*p* < 0.001) for *A. polyphaga*) and then increased up to 16 days (1.66 log CFU/ml increase (*p* < 0.05) for *A. castellanii* and 2.74 log CFU/ml increase (*p* < 0.05) for *A. polyphaga*), for both amoebae species (Figure 4). CFU counts were significantly higher in wells with insert than in wells without inserts at D16 (*p* < 0.05 both for *A. castellanii* and *A. polyphaga*) (Figure 4).

For the two bacterial strains, no bacteria were obtained from the amoebae supernatants lystates demonstrating that bacteria did not pass through the insert during the 16 days of the co-culture experiment.

### Immunofluoresence detection of *Francisella* in infected amoebae

**Figure 5.**
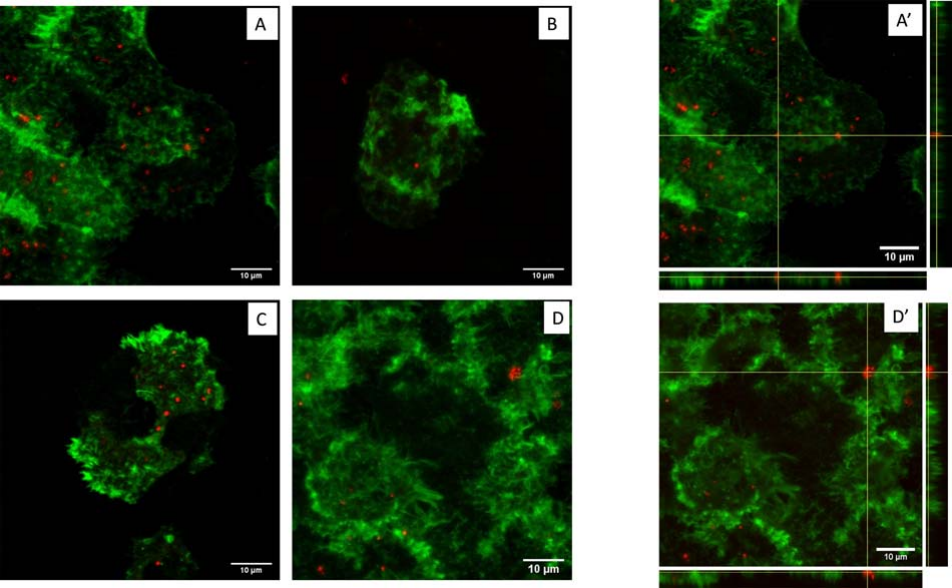
Confocal microscopy of *A. polyphaga* infected by *F. tularensis* Ft6. (A) T0 in PYG/wg; (B) D2 in PYG/wg; (C) D7 in PYG/wg; (D) T0 in SM; No more bacteria were observed inside amoeba at D7 in SM (data not shown). A’) orthogonal view of T0 in PYG/wg; D’) orthogonal view of T0 in SM. Green: actin (phalloidin Alexa Fluor 488). Red: *Francisella* sp. (rabbit primary antibody against *Francisella,* goat secondary antibody against rabbit Alexa Fluor 594).

Immunofluorescence staining and confocal microscopy were performed for the two amoebae species and the four *Francisella* strains at T0, D2 and D7 of infection in PYG/wg, and at T0 and D7 of infection in SM. Confocal microscopy confirmed that the four *Francisella* strains tested were located inside *A. polyphaga* or *A. castellanii* amoebae. No major variation was observed over time in PYG/wg, whereas bacteria disappeared at D7 in SM. Figure 5 shows confocal microscopy of *A. polyphaga* infected with *F. tularensis* Ft6, either in PYG/wg at T0, D2 and D7, or in SM at T0.

### Study of the T6SS impact on *Francisella* and amoebae interactions

In order to evaluate the role of the T6SS on *F. novicida* interactions with amoebae, we tested T6SS-deleted mutants compared to the wild-type (WT) U112 strain of *F. novicida* in the previously described models.

In APT model, *F. novicida* ΔFPI, *F. novicida* ΔFNI, and *F. novicida* ΔFPIΔFNI gave the same results with *A. castellanii* compared to the WT strain (Figure S1).

During infection in PYG/wg of *A. polyphaga*, the intra-amoebic counts of *F. novicida* WT and ΔFPIΔFNI strains evolved similarly although subtle but significant differences were observed at D2 and D7. There was a raise in CFU counts during two or five days, followed by a relative stabilization (Figure S3). Here again, CFU counts evolved similarly inside and outside amoebae, and were not significantly different between the mutant and WT strains over time (Figure S3).

During infection in SM of *A. polyphaga*, the intra-amoebic CFU counts of *F. novicida* WT and ΔFPIΔFNI decreased progressively and similarly over time despite bacterial counts slightly higher for *F. novicida* ΔFPIΔFNI (Figure S3).

Finally, during co-culture without inserts, *A. polyphaga* enhanced the survival of either *F. novicida* WT or *F. novicida* ΔFPIΔFNI in a similar manner. In contrast, the bacterial count of these two strains progressively and similarly decreased over time in amoeba-free medium (Figure S3).

### Amoebae and *Francisella* co-culture in spring water

To better mimic the natural conditions of *Francisella* sp. survival in aquatic environments, *F. novicida* U112 and *A. castellanii* co-cultures were performed in spring water and at room temperature, during 30 days.

**Figure 6.**
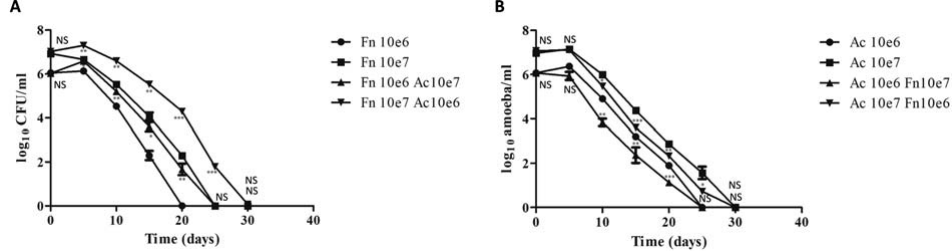
Growth kinetics of *F. novicida* (A) and *A. castellanii* (B) in spring water. The figure shows one experiment made in triplicate. Similar results were obtained in a second experiment. The error bars represent standard deviations. Comparison at each time point of (A) *F. novicida* with or without amoebae (B) *A. castellanii* with or without bacteria. NS: not significant; *: *p* < 0.05; **: *p* < 0.01; ***: *p* < 0.001. Fn: *F. novicida*; Ac: *A. castellanii*

In co-culture with amoebae, *F. novicida* showed enhanced survival compared to amoeba-free water samples, regardles of doses. During the observed period, bacterial counts in co-culture were higher in comparison to samples with no amoebae (*p* < 0.05 at D5, D10, D15, and D20, NS at D25 and D30 for *F. novicida* at 10^6^ CFU/ml and *p* < 0.01 at D5, D10, and D15, *p* < 0.001 at D20, *p* < 0.01 at D25 and NS at D30 for *F. novicida* at 10^7^ CFU/ml) (Figure 6(A)). Moreover, *F. novicida* survived for a longer period of time in the presence of amoebae.

Unlike previous models, in the spring water co-culture model, amoebic counts were not stable during the experiments and decreased progressively. Besides, in co-culture models, amoebic counts decreased slightly faster in comparison to control water samples with no bacteria (NS at D5, *p* < 0.01 at D10, *p* < 0.01 at D15, *p* < 0.001 at D20, NS at D25 and D30 for *A. castellanii* at 10^6^ cells/ml and NS at D5, *p* < 0.05 at D10, *p* < 0.001 at D15, *p* < 0.01 at D20, *p* < 0.05 at D25 and NS at D30 for *A. castellanii* at 10^7^ cells/ml) (Figure 6(B)).

**Figure 7.**
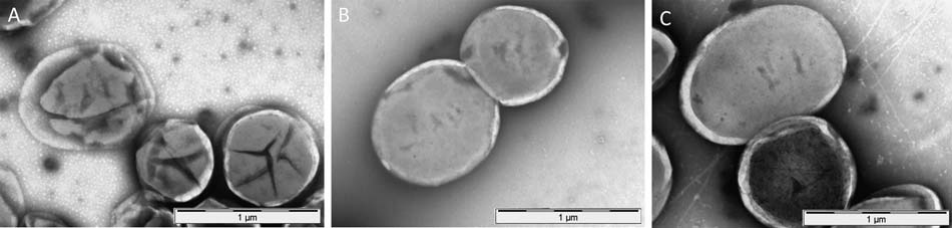
Morphology and structure of *F. novicida*, after 15 days incubation in spring water (A), co-incubation with *A. castellanii* in spring water (B), and as a control after growth on BCYE agar (C) by TEM.

Morphology of *F. novicida*, after 15 days incubation alone in spring water or in co-incubation with *A. castellanii* in spring water was also studied by transmission electron microscopy (TEM). Multiple morphological changes of *F. novicida* cells were observed after 15 days incubation in amoeba-free spring water. Bacteria showed disorganized cytoplasm separated from the cell wall, with multiple clumping and highly undefined cell wall. In contrast, *F.novicida* co-cultured with amoebae in spring water were roundly shaped and showed well preserved cell structures with smooth and intact cell wall, similar to the control bacteria grown on BCYE agar (Figure 7).

## Discussion

The aim of our study was to evaluate *in vitro* survival or even proliferation of *Francisella* species in the presence of amoebae over an extended period. This could help to understand their long-term survival in natural aquatic environments. Therefore, we set-up different models of interactions between *F. tularensis*, *F. philomiragia*, or *F. novicida* with two amoeba species, *A. castellanii* and *A. polyphaga*.

We first evaluated the interactions between *Francisella* and amoebae using the Amoeba Plate Test (APT). This screening test was developed by Albers *et al*. [24] to study the interactions between *L. pneumophila* and amoebae [24]. The APT evaluates the ability of a specific bacterial strain to pass through a layer of amoebae to form colonies on an agar plate. A negative APT usually indicates that the tested bacterium is unable to kill amoebae either directly or through intra-amoebic multiplication. Conversely, a positive test usually indicates that the bacterium is able to resist the attack by amoebae and possibly multiply within these protozoa [6]. Interestingly, we observed a positive APT for all tested *Francisella* strains but with varying results. We observed a strong growth with *F. philomiragia,* a medium growth with *F. novicida* and the clinical strains of *F. tularensis*, and the weakest growth with the LVS strain. As for *F. philomiragia*, our results are in agreement with those reported by Verhoeven *et al.* [10] showing higher resistance of *F. philomiragia* compared to *F. novicida* in an *A. castellanii* APT model and those of Thelaus *et al.* [25] showing that *F. philomiragia* was the least edible *Francisella* species for the ciliate *Tetrahymena pyriformis*. Regarding *F. tularensis* subsp. *holarctica* strains, LVS was less efficient to resist to amoebae than the clinical strains. This might be due to the attenuated virulence of this vaccine strain. Interestingly, the six clinical strains of *F. tularensis* subsp. *holarctica* displayed similar results, suggesting a homogenous amoeba resistance between strains of this subspecies, correlating with the low genetic diversity of French Type B strains [22]. Overall, the APT demonstrated some interactions between *Francisella* sp. and amoebae, with variations between *Francisella* species that could suggest different degree of adaptation to protozoan predation, as previously suggested by Thelaus *et al.* [25].

To better characterize interactions between *Francisella* and amoebae, we then used different cell models: 1/ an infection model in rich culture medium previously described in the literature [9,11] but with longer incubation period; 2/ an infection model in poor culture medium developed to prevent any extracellular growth of *Francisella*; and 3/ co-culture models in which amoebae and *Francisella* were allowed to interact through direct contact or not.

We first evaluated the ability of *Francisella* to multiply in amoeba culture media (PYG, PYG/wg, SM and PAS) in the absence of these protozoa. In models evaluating the ability of bacteria to grow inside eukaryotic cells, the culture supernatant should not support their extracellular growth. *F. philomiragia*, *F. novicida,* and *F. tularensis* Ft6 and LVS strains displayed a strong growth in PYG and PYG/wg. This observation was previously reported by El-Etr *et al.* [8]. Because we incubated our cultures for up to 16 days, we considered that an extracellular growth of bacteria may lead to erroneous results. Thus, in our experiments, we did not use the PYG and PYG/wg media but rather the SM medium for which no bacterial growth was observed.

In the infection in PYG/wg model, use as control, we observed a two- to four-log increase in CFU counts of *F. novicida*, *F. philomiragia* and Ft6 strains within amoebae during the first two or five days of infection (Figure 1). Confocal microscopy confirmed that bacteria were present inside the amoebae (Figure 5). These findings were similar to those previously reported by Santic *et al.* [9] for *F. novicida* and *A. castellanii* or *V. vermiformis*. Yet, the next days of the experiments we observed a stagnation or reduction in intra-amoebic CFU counts. However, extracellular CFU counts overlaid intracellular CFU counts. The LVS strain did not exhibit any CFU increase in the intra- or extra-amoebic compartments, while this bacterium was able to grow in amoeba-free PYG/wg. This result suggests that the LVS strain was phagocytosed and digested by the amoebae, which may be correlated to the low virulence of this vaccine strain. Because of extra-amoebic growth of most *Francisella* strains tested, we considered this model not suitable for long-term evaluation of the intra-amoebic growth of these bacteria.

The infection in SM model was designed to allow only intra-amoebic growth of *Francisella* strains, by using SM as the culture medium. The SM supported the survival of *A. castellanii* and *A. polyphaga* for 16 days in trophozoite forms (data not shown), but did not allow growth of the tested *Francisella* sp. strains (Figure S2). In this model, no intra-amoebic replication of *F. novicida*, *F. philomiragia*, LVS and Ft6 was observed. A rapid decrease of intra-amoebic CFU counts was observed (Figure 2). Confocal microscopy confirmed that *Francisella* sp. were localised inside the amoebae at T0, but then disappeared at D7 post infection (Figure 5), a result correlating with reduction in CFU counts. El-Etr *et al.* also reported a decrease in intracellular counts of LVS and some clinical strains of *F. tularensis* subsp. *tularensis*, in an infection model using a poor culture medium (High-salt buffer) [8]. However, in the same study, an increase in intra-amoebic CFU counts was observed for *F. novicida* U112 and others *F. tularensis* subsp. *tularensis* clinical strains [8] suggesting that intra-amoebic growth could depend on the *Francisella* species and strain studied and also on experimental protocol used.

Since APT revealed that *Francisella* sp. resisted to amoebae and infection in SM did not suggested intra-amoebic survival of *Francisella* sp., we developed co-culture models. The aim of the co-culture model was to evaluate if amoebae could promote *Francisella* sp. survival whether intracellular or extracellular. Enhanced survival of *F. philomiragia* and *F. novicida* was observed in the presence of amoebae, while we found a rapid loss of cultivability of these bacteria alone in amoeba-free medium (Figure 3). Buse *et al.* also showed that several amoebae species enhanced bacterial survival but did not enable multiplication of *F. novicida*, LVS and type-A and type-B clinical strains [14]. Unlike this study, we observed a real multiplication of both *F. novicida* and *F. philomiragia* after seven to 12 days of incubation (Figure 3). However, Buse *et al.* stopped there experimentations after 10 days of co-incubation, maybe just before bacterial multiplication onset [14]. This suggested that amoebae could promote the survival of these two *Francisella* species. Two mechanisms should be considered to explain this enhanced survival in the presence of amoebae: 1/ a direct “physical” interaction between bacteria and amoebae; 2/ the production of nutritive elements by amoebae in the culture supernatant that could be useful for *Francisella* survival and/or a degradation of toxic compounds by amoebae. In order to investigate these two hypotheses, we repeated the same co-culture experiments with the addition of inserts to physically separate bacteria from amoebae while allowing them to share the same medium. Enhanced survival of *F. novicida* was partially lost when an insert separated amoebae and bacteria (Figure 4). This result suggested that enhanced survival of *F. novicida* in co-culture with amoebae could be partly dependent on direct contact between bacteria and protozoa but also on amoeba-excreted compounds. Experiments with *F. philomiragia* gave too heterogeneous results within and between experiments to draw any conclusion.

Although the Ft6 strain did not survive in amoeba co-cultures without insert, we observed an enhanced survival of this strain when separated from the amoebae (Figure 4). We can hypothesize that, because *F. tularensis* strains (including Ft6) have a lower growth rate than *F. philomiragia* and *F. novicida*, phagocytosis by amoebae may outcompete bacterial growth in co-culture models without insert leading to a reduction in bacterial CFU counts. In contrast, when amoebae cannot phagocytize Ft6 because of the insert, this bacterium may benefit from amoeba-excreted compounds. This differ slightly from the observations of Buse *et al.* [14] who reported an enhanced survival of a *F. tularensis* type B strain in contact with amoebae, which may indicate that results are dependent on the bacterial strain used or on the experimental protocol. Verhoeven *et al.* reported that the inoculation of *F. philomiragia* ATCC 25015 in PYG medium preconditioned by *A. castellanii* (i.e. growth supernatant of *A. castellanii* from which the amoebae were removed) reduced biofilm production and increased bacterial growth [10]. Gustafsson *et al.* also demonstrated the same positive effect of culture media preconditioned by *A. palestinensis* on the growth of *F. tularensis* LVS [26]*.* These two studies suggested that several *Francisella* species could benefit from elements excreted by amoebae during co-culture with these protozoans.

Co-culture models of *F. novicida* with *A. castellanii* were then performed in a spring water environment and at room temperature to be as close as possible from natural ecosystem. In this model, amoebae again favoured *F. novicida* survival. Indeed, bacterial counts were higher in the presence of amoebae compared to amoeba-free spring water and survival time of *F. novicida* was longer in the presence of amoebae (Figure 6(A)). In addition, bacterial morphology was preserved in presence of amoebae compared to amoeba-free spring water (Figure 7).

In all the models tested, we did not observe any difference between WT *F. novicida* and a *F. novicida* strain deleted of the two loci encoding T6SS, strongly suggesting that *F. novicida* does not rely on its T6SSs to interact with amoebae. This result is in sharp contrast with the key role of the FPI-encoded T6SS in promoting bacterial replication in mammalian cells [17]. However, this feet well with our infection and co-culture experiments results that suggest an absence of *Francisella* sp. replication inside amoebae but a *Francisella* sp. survival and multiplication in the presence of amoebae, probably extracellularly.

Overall, data available in the literature on the interactions between *Francisella* sp. and amoebae are conflicting. Some studies described the multiplication of *F. noatunensis*, *F. philomiragia*, *F. novicida*, LVS, and clinical strains of *F. tularensis* subsp. *tularensis* inside or in association with amoebae such as *A. castellanii*, *V. vermiformis* and *Dictyostelium discoideum* [8–13]. Conversely, other publications reported a lack of multiplication of *F. novicida*, LVS or type A and type B clinical strains [8,14]. However, these studies used very different experimental protocols, especially in terms of amoebae culture media. Previous works reporting multiplication of *Francisella* strains inside or in the presence of amoebae for 12 or 20 days have used rich media such as PYG medium (ATCC medium 712) [10,11]. For such models, it may be difficult to distinguish intra- from extra-amoebic growth of bacteria, and increase in CFU counts should be interpreted cautiously. To overcome this potential bias, we decided to work in SM medium, which did not support growth of *Francisella* strains used in this study. We evaluated for the first time the interactions of amoebae with several virulent clinical strains of *F. tularensis* subsp. *holarctica.* Although more tedious, these models can be considered more relevant to study ecological aspects of this major human pathogen compared to the attenuated LVS strain or strains belonging to other *Francisella* species. Hence, very different results were obtained in this study between the studied *Francisella* species, but also between LVS and clinical strains of *F. tularensis* subsp. *holarctica*.

In our models, *F. philomiragia*, *F. novicida* and virulent type B strain of *F. tularensis* did not multiply inside amoebae, but could survive and multiply in an environment containing amoebae suggesting a potential commensal relationship between these two microorganisms. The interaction conditions established in our co-culture models likely occur in the aquatic environment where amoebae are widespread [7]. We found that *F. novicida* and *F. philomiragia* survive in the presence of amoebae. These two species are considered to have a primary aquatic reservoir, which fits well with the water-borne nature of the rare human infections caused by these two pathogens [5]. We also demonstrated that fully virulent *F. tularensis* strains may benefit from the presence of amoebae to survive in the environment. This may partly explain why human tularemia cases are occasionally associated with water-born transmission [5]. However, the variable ability of *Francisella* species to interact with amoebae, as described in the literature, also suggests that amoebae should not be considered the only reservoir of these bacteria in the aquatic environment. Other mechanisms potentially involved may include survival of *Francisella* sp. in biofilms, in mosquitoes larvae, and in water as viable but non-culturable (VBNC) forms [5] and have to be further investigated.

## Supplementary Material

Table_S1_final.docx

Figure_S3_final.tif

Figure_S2_final.tif

Figure_S1_final.tif

SUPPLEMENTARY_FIGURES_LEGENDS_final.docx

## References

[1] Dennis DT, Inglesby TV, Henderson DA, et al. Tularemia as a biological weapon: medical and public health management. JAMA. 2001;285:2763–2773. 11386933 10.1001/jama.285.21.2763

[2] Johansson A, Celli J, Conlan W, et al. Objections to the transfer of *Francisella novicida* to the subspecies rank of *Francisella tularensis*. Int J Syst Evol Microbiol. 2010;60:1717–1718. author reply 1718-1720. 20688748 10.1099/ijs.0.022830-0 PMC7442299

[3] Busse H-J, Huber B, Anda P, et al. Objections to the transfer of *Francisella novicida* to the subspecies rank of *Francisella tularensis* - response to Johansson et al. Int J Syst Evol Microbiol. 2010;60:1718–1720. 20688749 10.1099/00207713-60-8-1718

[4] Sjöstedt A. Tularemia: history, epidemiology, pathogen physiology, and clinical manifestations. Ann N Y Acad Sci. 2007;1105:1–29. 17395726 10.1196/annals.1409.009

[5] Hennebique A, Boisset S, Maurin M. Tularemia as a waterborne disease: a review. Emerg Microbes Infect. 2019;8:1027–1042. 31287787 10.1080/22221751.2019.1638734 PMC6691783

[6] Greub G, Raoult D. Microorganisms resistant to free-living amoebae. Clin Microbiol Rev. 2004;17:413–433. 15084508 10.1128/CMR.17.2.413-433.2004 PMC387402

[7] Rodríguez-Zaragoza S. Ecology of free-living amoebae. Crit Rev Microbiol. 1994;20:225–241. 7802958 10.3109/10408419409114556

[8] El-Etr SH, Margolis JJ, Monack D, et al. *Francisella tularensis* type A strains cause the rapid encystment of *Acanthamoeba castellanii* and survive in amoebal cysts for three weeks postinfection. Appl Environ Microbiol. 2009;75:7488–7500. 19820161 10.1128/AEM.01829-09 PMC2786426

[9] Santic M, Ozanic M, Semic V, et al. Intra-vacuolar proliferation of *F. novicida* within *H. vermiformis*. Front Microbiol. 2011;2:78. 21747796 10.3389/fmicb.2011.00078 PMC3128938

[10] Verhoeven AB, Durham-Colleran MW, Pierson T, et al. *Francisella philomiragia* biofilm formation and interaction with the aquatic protist *Acanthamoeba castellanii*. Biol Bull. 2010;219:178–188. 20972262 10.1086/BBLv219n2p178

[11] Lauriano CM, Barker JR, Yoon S-S, et al. Mgla regulates transcription of virulence factors necessary for *Francisella tularensis* intraamoebae and intramacrophage survival. Proc Natl Acad Sci USA. 2004;101:4246–4249. 15010524 10.1073/pnas.0307690101 PMC384726

[12] Lampe EO, Brenz Y, Herrmann L, et al. Dissection of *Francisella*-host cell interactions in *Dictyostelium discoideum*. Appl Environ Microbiol. 2015;82:1586–1598. 26712555 10.1128/AEM.02950-15 PMC4771330

[13] Abd H, Johansson T, Golovliov I, et al. Survival and growth of *Francisella tularensis* in *Acanthamoeba castellanii*. Appl Environ Microbiol. 2003;69:600–606. 12514047 10.1128/AEM.69.1.600-606.2003 PMC152416

[14] Buse HY, Schaefer Iii FW, Rice EW. Enhanced survival but not amplification of *Francisella* spp. in the presence of free-living amoebae. Acta Microbiol Immunol Hung. 2017;64:17–36. 27929353 10.1556/030.63.2016.015 PMC7357732

[15] Gray CG, Cowley SC, Cheung KKM, et al. The identification of five genetic loci of *Francisella novicida* associated with intracellular growth. FEMS Microbiol Lett. 2002;215:53–56. 12393200 10.1111/j.1574-6968.2002.tb11369.x

[16] Nano FE, Zhang N, Cowley SC, et al. A *Francisella tularensis* pathogenicity island required for intramacrophage growth. J Bacteriol. 2004;186:6430–6436. 15375123 10.1128/JB.186.19.6430-6436.2004 PMC516616

[17] Broms JE, Sjostedt A, Lavander M. The role of the *Francisella tularensis* Pathogenicity Island in type VI secretion, intracellular survival, and modulation of host cell signaling. Front Microbiol. 2010;1; doi:10.3389/fmicb.2010.00136. PMC3109350 21687753

[18] Eshraghi A, Kim J, Walls AC, et al. Secreted effectors encoded within and outside of the *Francisella* Pathogenicity Island promote intramacrophage growth. Cell Host Microbe. 2016;20:573–583. 27832588 10.1016/j.chom.2016.10.008 PMC5384264

[19] Rigard M, Broms JE, Mosnier A, et al. *Francisella tularensis* IglG belongs to a novel family of PAAR-like T6SS proteins and harbors a unique N-terminal extension required for virulence. PLoS Pathog. 2016;12, e1005821. 10.1371/journal.ppat.1005821 PMC5014421 27602570

[20] Köppen K, Chen F, Rydzewski K, et al. Screen for fitness and virulence factors of *Francisella* sp. strain W12-1067 using amoebae. Int J Med Microbiol. 2019;309, 151341. 10.1016/j.ijmm.2019.151341 31451389

[21] Weiss DS, Brotcke A, Henry T, et al. *In vivo* negative selection screen identifies genes required for *Francisella* virulence. Proc Natl Acad Sci USA. 2007;104:6037–6042. 17389372 10.1073/pnas.0609675104 PMC1832217

[22] Kevin M, Girault G, Caspar Y, et al. Phylogeography and genetic diversity of *Francisella tularensis* subsp. *holarctica* in France (1947-2018). Front Microbiol. 2020;11:287. 32194525 10.3389/fmicb.2020.00287 PMC7064806

[23] Reteno DG, Benamar S, Khalil JB, et al. Faustovirus, an asfarvirus-related new lineage of giant viruses infecting amoebae. J Virol. 2015;89:6585–6594. 25878099 10.1128/JVI.00115-15 PMC4468488

[24] Albers U, Reus K, Shuman HA, et al. The amoebae plate test implicates a paralogue of lpxB in the interaction of *Legionella pneumophila* with *Acanthamoeba castellanii*. Microbiology (Reading, Engl). 2005;151:167–182. 10.1099/mic.0.27563-0 15632436

[25] Thelaus J, Andersson A, Mathisen P, et al. Influence of nutrient status and grazing pressure on the fate of *Francisella tularensis* in lake water. FEMS Microbiol Ecol. 2009;67:69–80. 19120459 10.1111/j.1574-6941.2008.00612.x

[26] Gustafsson K. Growth and survival of four strains of *Francisella tularensis* in a rich medium preconditioned with *Acanthamoeba palestinensis*. Can J Microbiol. 1989;35:1100–1104. 2630032 10.1139/m89-184

