# Supplementary material for: Amoebae can promote the survival of Francisella species in the aquatic environment
Source: Emerg Microbes Infect. 2021 Feb 24;10(1):277–90. doi: 10.1080/22221751.2021.1885999 (PMC7919924; doi:10.1080/22221751.2021.1885999)
Supplement: Table_S1_final.docx [file TEMI_A_1885999_SM1779.docx]

**Table S1. *Francisella* sp. strains used in this study**

| Strains | Source |
| --- | --- |
| *F. tularensis* subsp. *holarctica* LVS NCTC 10857 | Reference strain given by the CRSSA, Grenoble, France |
| *F. tularensis* subsp. *holarctica* Ft5 | Human, blood (France, 2004) |
| *F. tularensis* subsp. *holarctica* Ft6 | Human, blood (France, 2007) |
| *F. tularensis* subsp. *holarctica* Ft7 | Human, conjunctiva (France, 2006) |
| *F. tularensis* subsp. *holarctica* Ft46 | Human, hear (France, 2014) |
| *F. tularensis* subsp. *holarctica* Ft62 | Human, lymphadenopathy (France, 2016) |
| *F. tularensis* subsp. *holarctica* Ft74 | Human, hip prosthesis (France, 2017) |
| *F. philomiragia* ATCC 25015 | Reference strain from the ATCC |
| *F. philomiragia* Ft47 | Human, blood (France, 2014) |
| *F. novicida* U112 (CIP 56.12) | Reference strain from the CIP |
| *F. novicida* Δ*FPI* | (Weiss *et al.*, 2007) |
| *F. novicida* Δ*FNI* | (Rigard *et al.*, 2016) |
| *F. novicida* Δ*FPI* Δ*FNI* | This study |

# NCTC: National Collection of Type Cultures (England); ATCC: American Type Culture Collection (USA); CIP: Collection de l’Institut Pasteur (France)
