# Supplementary figures and images for: Amoebae can promote the survival of Francisella species in the aquatic environment
Source: Emerg Microbes Infect. 2021 Feb 24;10(1):277–90. doi: 10.1080/22221751.2021.1885999 (PMC7919924; doi:10.1080/22221751.2021.1885999)

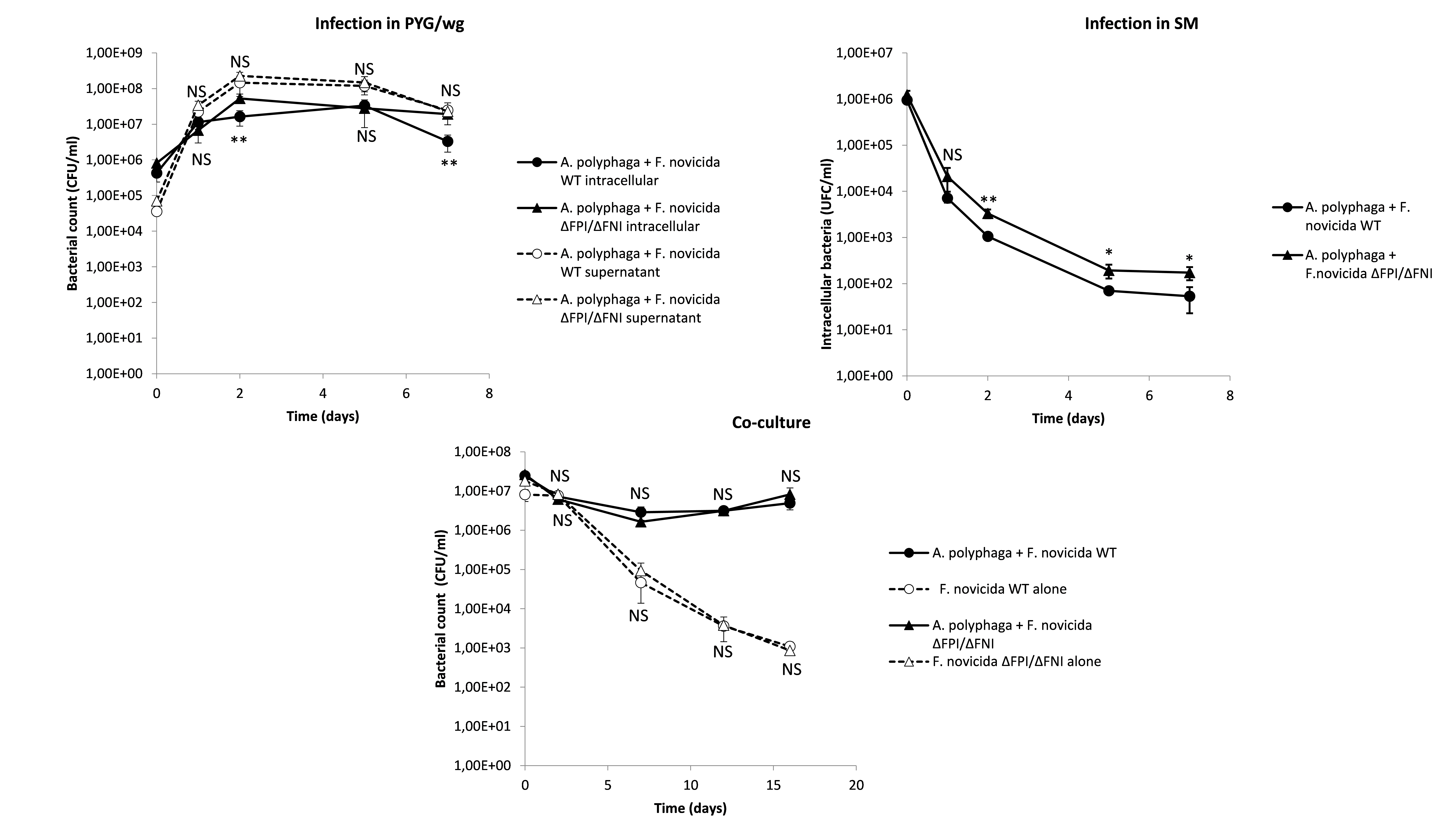

Supplement: Figure_S3_final.tif [file TEMI_A_1885999_SM1778.tif]

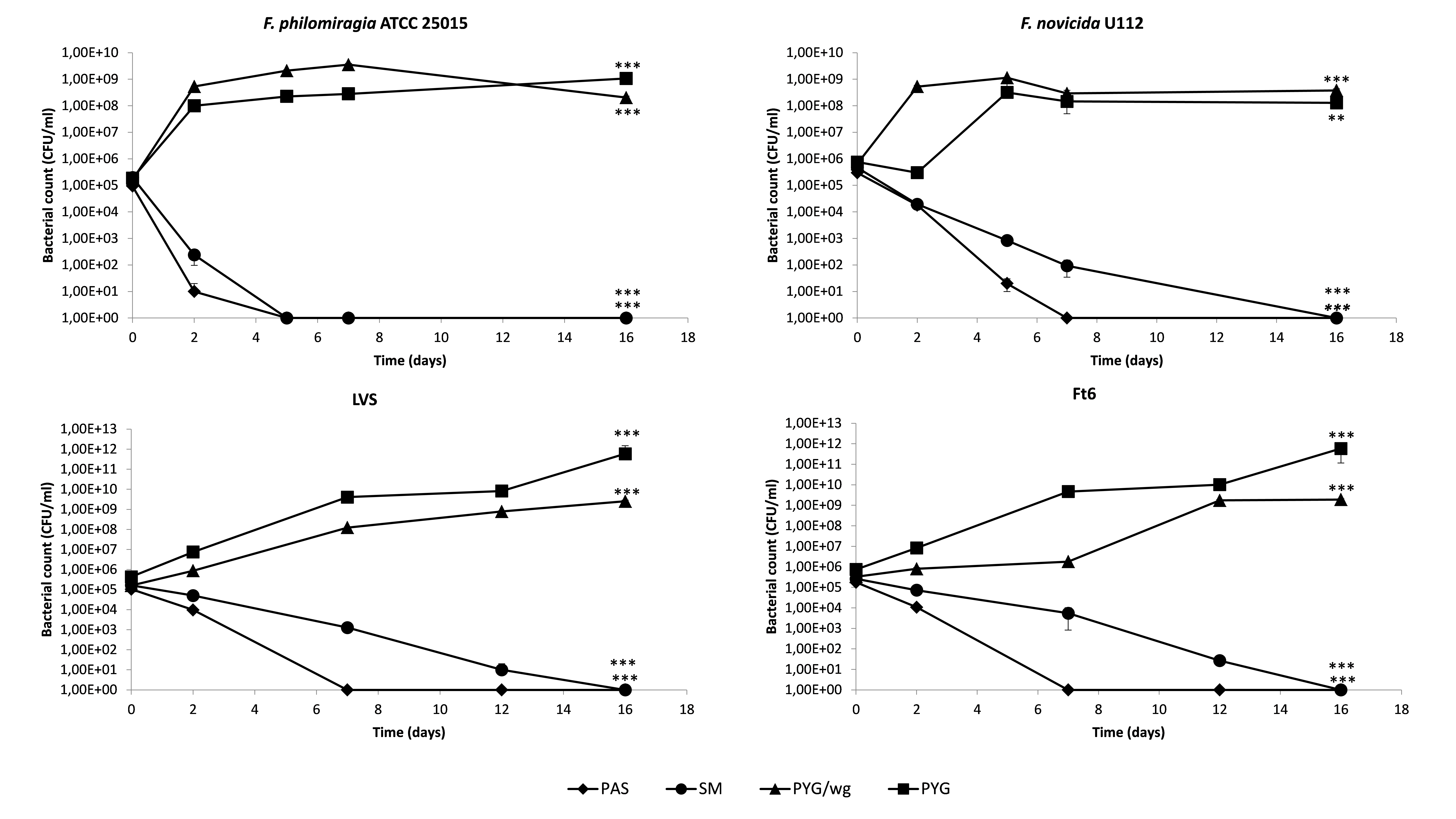

Supplement: Figure_S2_final.tif [file TEMI_A_1885999_SM1777.tif]

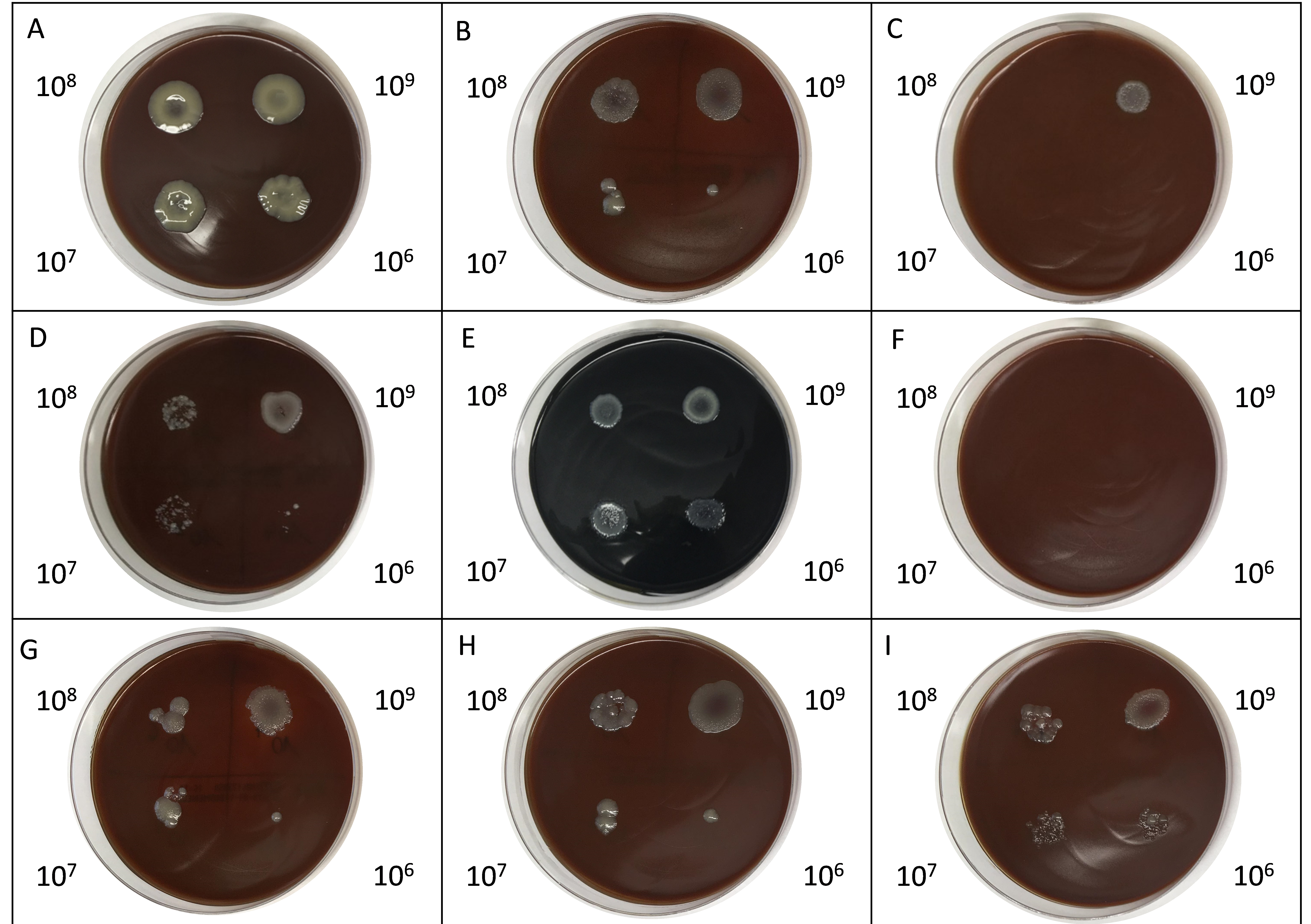

Supplement: Figure_S1_final.tif [file TEMI_A_1885999_SM1776.tif]
