# Supplementary material for: Amoebae can promote the survival of Francisella species in the aquatic environment
Source: Emerg Microbes Infect. 2021 Feb 24;10(1):277–90. doi: 10.1080/22221751.2021.1885999 (PMC7919924; doi:10.1080/22221751.2021.1885999)
Supplement: SUPPLEMENTARY_FIGURES_LEGENDS_final.docx [file TEMI_A_1885999_SM1775.docx]

**SUPPLEMENTARY FIGURES LEGENDS**

**Figure S1. Example of Amoeba Plate Test results of *Acanthamoeba castellanii* and several bacteria at day 9**

A) *Francisella philomiragia* ATCC 25015. B) *F. novicida* U112. C) *F. tularensis* LVS. D) *F. tularensis* Ft6. E) *Legionella pneumophila* CIP107629T. F) *Staphylococcus epidermidis* ATCC 1228. G) *F. novicida* ∆FPI. H) *F. novicida* ∆FNI. I) *F. novicida* ∆FPI∆FNI.

**Figure S2. Extracellular growth of *Francisella* sp. in several culture media**

The figure shows one experiment made in triplicate. Similar results were obtained in a second experiment. The error bars represent standard deviations. Comparison of T0 versus D16; NS = not significant; *: p<0.05; **: p<0.01; ***: p<0.001

**Figure S3. Study of T6SS impact in three models.**

The figure shows one experiment made in triplicate. Similar results were obtained in a second experiment. The error bars represent standard deviations. Comparison of *F. novicida* WT and *F. novicida* ∆FPI∆FNI bacterial counts over time. NS: not significant; *: p < 0.05; **: p < 0.01; ***: p < 0.001
